# Dynamic Modeling of Prevention and Control of *Brucellosis* in China: A Systematic Review

**DOI:** 10.1155/tbed/1393722

**Published:** 2025-01-11

**Authors:** Liu Yang, Meng Fan, Youming Wang

**Affiliations:** ^1^Epidemiological Investigation Department, China Animal Health and Epidemiology Center, Qingdao 266032, Shandong, China; ^2^National Center for Applied Mathematics in Jilin, Center for Mathematical Biosciences, School of Mathematics and Statistics, Northeast Normal University, 5268 Renmin Street, Changchun 130024, Jilin, China

**Keywords:** brucellosis, dynamic model, measure evaluation, parameter estimation, reproduction number

## Abstract

Brucellosis is a zoonotic infectious disease caused by bacteria of the genus Brucella. In recent years, the prevalence of brucellosis in animals and humans has been increasing in China despite the considerable efforts taken to date. Dynamic model serves as an influential and promising approach for offering guidance and recommendations for the prevention and control of the disease. At this pivotal moment, it is time to provide a comprehensive and timely examination of the existing achievements derived from the mathematical dynamical modeling studies, highlight the key development trends, delve into identifying the limitations of the studies, and offer valuable perspectives and insights for potential future research directions. Through a review of 49 articles (22 articles utilizing data while 27 articles did not use data), this study focuses on analyzing the differences in model structure, research data and areas, characterization of prevention and control measures, and main results. Meanwhile, quantitative results such as the reproduction number and critical parameter values are extracted. The study points out that the limitations of existing models are manifested in the lack of heterogeneity in the research and the absence of the results on the scale of herd/flock. The primary reason is the lack of relevant data, indicating the necessity to advance interdisciplinary, multidisciplinary, and transdisciplinary collaboration across multiple departments. Therefore, it is encouraged that the future models should be established from the holistic approach of One Health.

## 1. Introduction

**Figure 1:**
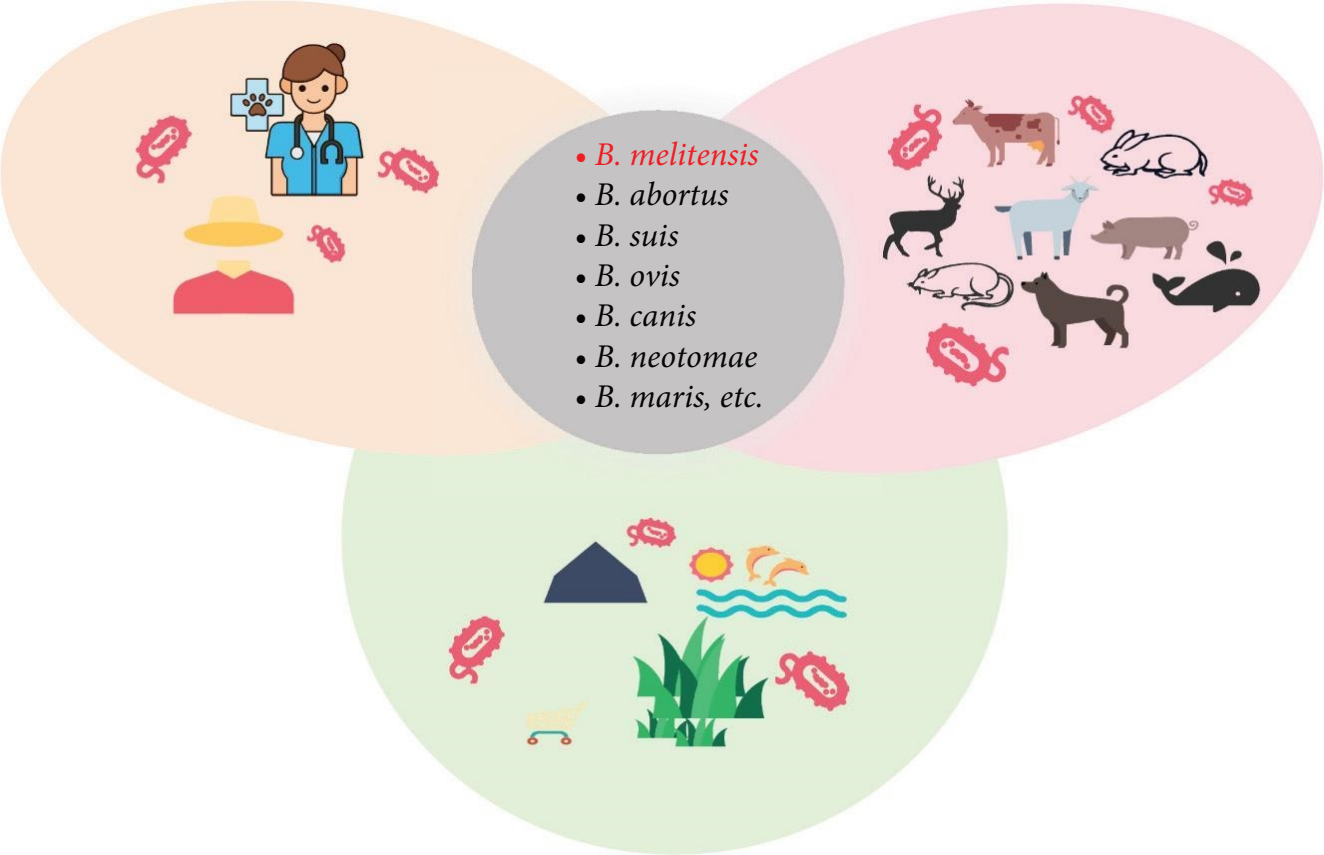
Brucellosis transmission from a perspective of “One Health.” Notice that the listed bacterial strains are not all zoonotic.

Brucellosis (also known as “undulant fever,” “Mediterranean fever,” or “Malta fever”) is a bacterial zoonosis caused by various Brucella species, for example, *B. melitensis*, *B. abortus*, *B. suis*, *B. canis*, etc. [1], which mainly infect livestock including cattle, swine, goats, sheep, and dogs. Humans are infected with the disease by direct contact with infected animals or by indirect contact with the pathogen in the environment (find more details in Figure 1). Brucella can survive in the environment for months [1]. Government and health agencies worldwide have made concerted efforts to prevent and control the spread of brucellosis. However, brucellosis remains a neglected zoonotic disease and its prevention and control are very challenging in many countries [2–4]. Brucellosis poses a serious threat to animal health, human health, and environmental health. In contrast, human behavior, social factors, and environmental factors also affect the transmission of the disease [5]. It is reasonable and realistic to consider the disease transmission from the perspective of One Health [6].

Human brucellosis still remains a major public health concern in mainland of China. The incidence of human brucellosis exhibits a general upward trend from 2004 to 2023, with an increase tendency from 2004 to 2015, then followed by a gradual decline from 2016 to 2018, and another significant increase from 2019 to 2023 [7]. Human brucellosis has experienced a resurgence in China and is characterized by a noticeable and notable spatiotemporal shift in its geographic distribution, with a consistent high incidence in the northern regions and with a steady increase in the western and southern regions (i.e., nonpastoral areas) [5, 8]. Most human infections are farmers and herders while urban populations are also at risk of infection with the progress of urbanization. As the scale of animal husbandry expands, brucellosis also poses a serious hazard to livestock production [9].

The Chinese government has been working in recent years to establish the brucellosis-free areas where the disease should be eliminated for several years [10]. Vaccination and testing as well as slaughter are two vital means during the elimination of brucellosis [11]. Meanwhile, some complementary measures are also applied, such as proper hygiene practices, disinfection protocols, controlled access to facilities, etc. Chinese government adopts the strategy of prevention and control by regions. Vaccination measure is recommended for some serious regions, while testing and slaughter are recommended for other regions [12]. In practice, although the term “high-risk regions” is mentioned in government documents, it is actually very difficult to accurately define “high-risk regions” and to find the threshold conditions for switching of control measures. Therefore, there are still some urgent problems to be solved during the prevention and control of the disease.

Modeling study plays a crucial role in the prevention and control of livestock diseases, such as foot and mouth disease [13, 14], African swine fever [15], avian influenza [16], and so on. With the development of modeling studies, more and more models are being extensively used, which can be broadly divided into statistical models, computer models, and dynamic models. It should be noticed that these models are not mutually exclusive, and some knowledge is overlapping. Statistical models, including the autoregressive integrated moving average (ARIMA) model [17–19], the eXtreme Gradient Boosting (XGBoost) model [17, 20], regression model [21], and so on, are utilized to explore the relationship between driving factors and the prevalence of diseases, and also to predict the disease transmission. Computer models involving agent-based models, neural network models, and so on, achieve the computation and simulation of complex systems [22, 23]. Both of those two types of modeling studies require a considerable amount of data. Dynamic models are also numerous, and their advantage is that the model can be established based on mechanisms, thus they can be applicable even when there is limited data.

Mathematical–dynamic modeling approach has been extensively applied in population dynamics [24, 25] and infectious disease dynamics [26]. Dynamic model for brucellosis transmission and control has been applied since the 1970s in some countries [27–30]. Epidemiological models involve many classifications in terms of different characteristics. Hurd and Kaneene [29] identified three general types of models, chain binomial models, mass action models, and system models, where mass action models belong to dynamic models and are established based on the mechanism of the disease transmission. When data information is incomplete, transmission dynamic model helps to provide valuable insights into understanding disease dynamics, assessing risk factors, evaluating policy impacts, and supporting surveillance efforts. When the data information is comprehensive, dynamic model can be used to predict disease outbreaks and optimize control strategies.

In terms of the high prevalence of the disease and the development of dynamic models, it is time to summarize existing results, identify shortcomings, and provide insights for the future work. In Section 2, the materials and methods are presented.  Section 3 expounds the main results from the reviewing literature. Some confusions on the modeling are discussed in Section 4. In Section 5, the paper ends with some illuminating conclusions. The details of the searched papers are listed in Supporting Information section.

## 2. Materials and Methods

The systematic review was conducted in accordance with the guidelines for preferred reporting items for systematic reviews and meta-analysis (PRISMA) [31].

### 2.1. Survey Methodology

First of all, the modeling studies applied to brucellosis are identified using the search items “brucellosis model,” “brucellosis modeling,” “brucellosis modeling,” and “China” in Google Scholar. In the Web of Science database, “brucellosis, model, China,” “brucellosis, modeling, China,” and “brucellosis, modeling, China” in topic are searched. As of April 29, 2024, 234 entries and 114 entries are involved in Google Scholar and Web of Science, respectively.

### 2.2. Inclusion and Exclusion Criteria

**Figure 2:**
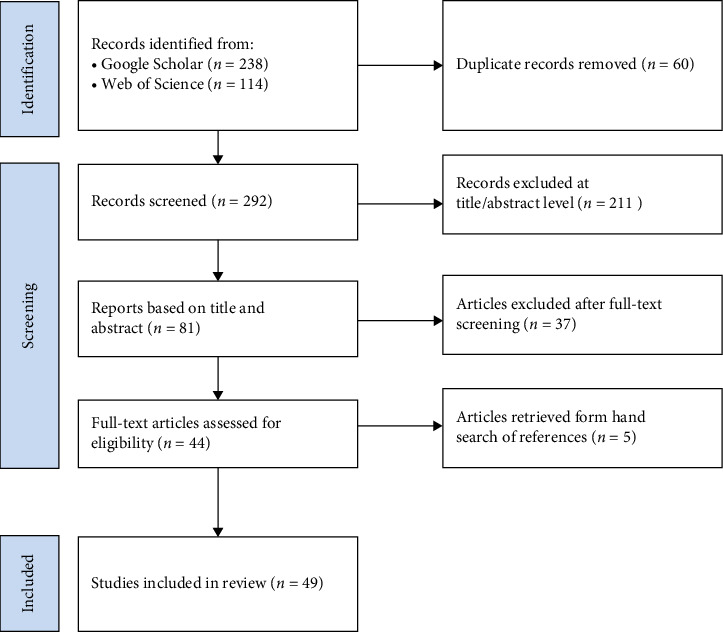
PRISMA flow diagram of the article selection process.

From the information of authors and article titles, 60 duplicate records are first discarded. Next, the studies, which do not apply mathematical models at the title and abstract level, are excluded. Finally, some articles by screening the full-text in terms of the following strategies are included: (1) the researches that mainly focus on dynamic models; (2) the literature that excludes the overlap between the review article and the regular paper; (3) the studies using data for China. Forty-nine articles in total are considered consistent and are included in this review (see Figure 2 and Supporting Information) together with one more review paper on dynamic model [8].

### 2.3. Data Extraction and Analysis

The key features of all selected articles are systematically extracted independently by the authors. By reviewing the articles, there are 22 articles combined with the data which are called the applied studies (see Table S2), and the other 27 articles focus on the mathematical results without data which are referred to as theoretical studies (see Table S1). For the applied studies, the information included study scope, model structure, control measures, data, reproduction number, and effect factors (see Tables 1 and S2). The other researches focused on the model structure and main findings (see Table S1).

### 2.4. Evaluation of Data Quality

In this subsection, the data quality of 22 applied studies is evaluated. Among them, six articles involve the information on livestock brucellosis, while the other 16 articles involve human brucellosis (see Table S2). By reviewing the sources of data information in the articles, the main foundations of human-reported cases are from the National Population and Health Science Data [54] sharing platform administered by the Chinese Center for Disease Control and Prevention. Data information on livestock brucellosis are from the local centers for animal epidemic prevention and control, such as the positive rate of dairy cows in [33, 35], and the data on sheep brucellosis in Inner Mongolia [36]. Data on adult sheep in one study are from China Animal Health and Epidemiology Center [36], and the data from another study [43] are from the research study published in a Chinese journal [55]. It shows that the data source is reliable, and the data information on livestock brucellosis is sparse compared to that of human. Although the methods of monitoring and diagnosis are not pointed out in the published articles, according to its data sources, whether it is the national animal disease prevention and control department or the local ones, both of them follow the standard of diagnostic techniques for animal brucellosis GB/T 18646-2002 [56] and GB/T 18646-2018 [57] governed by Ministry of Agriculture and Rural Affairs of the People's Republic of China.

## 3. Results

### 3.1. Research Area and Data

**Figure 3:**
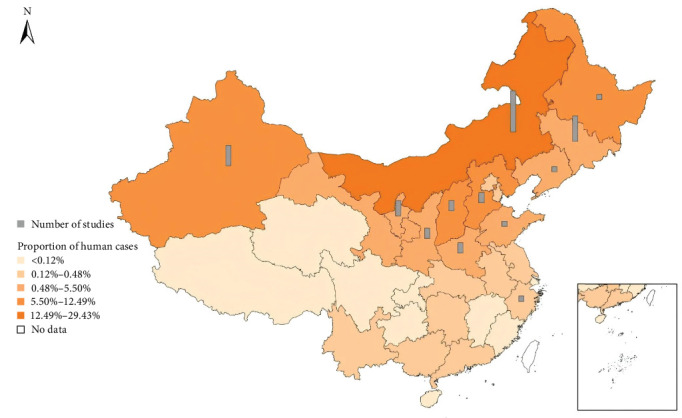
Regional distribution of existing studies and the proportion of infected cases in each region from 2004 to 2020. The data are from the Chinese Center for Disease Control and Prevention [58].

Since the number of applied studies is less than that of theoretical studies, there are still gaps between the current theoretical model and the real situation in most studies in China. From the scope of those studies, most of the researches take pastoral areas into account, such as Inner Mongolia [18, 32, 34], Xinjiang [38], Ningxia [49], and so on, while a few studies focus on agricultural areas (e.g., Zhejiang [33], Jilin [35], and so on). Little research has been done on semiagricultural and semipastoral areas, and Inner Mongolia, where the proportion of people with simultaneous infections is higher, has been studied more (see Figure 3).

Some parameter values can be obtained in terms of the social and environmental factors in the studied region, the pathogenic and epidemiological characteristics of the bacteria, and also the characteristics of the animals themselves. The recruitment rate of animals is seriously dependent on the studied regions and period, for example, it is taken as a mean value of 11,629,200 year^−1^ by the data from 2005 to 2010 in Inner Mongolia [32], and it is estimated about 15807 year^−1^ in Zhejiang Province by the data from 2001 to 2010 [33]. The average removal rate of animals (e.g., sheep, cows) is from 0.22 to 0.4 since the life span is about 4–5 years [32, 33] and the average removal period is 2.5 years. The testing sensitivity is about 85% [33, 34]. The natural mortality of brucella is 6 year^−1^ since it remains viable for about 2 months in the soil. The incubation period of brucellosis is about 2 weeks to several months [1, 59].

Some parameters being difficult to obtain in practice, such as transmission/incidence rates, initial values of infections, and so on, are estimated by data. The data used are human and animal infection cases. There are 16 articles in the applied studies applying data from human cases, and the others (6/22) utilize the data of the infection cases among livestock (see Table S2). In recent years, the effects of the economy on the spread of brucellosis have been explored and the data on price changes of livestock have also been used [50]. The fact that the data of human infections is more readily available than that of livestock makes the use of human cases more common. On this basis, the trend of model solutions (i.e., infection cases) can be well simulated.

**Figure 4:**
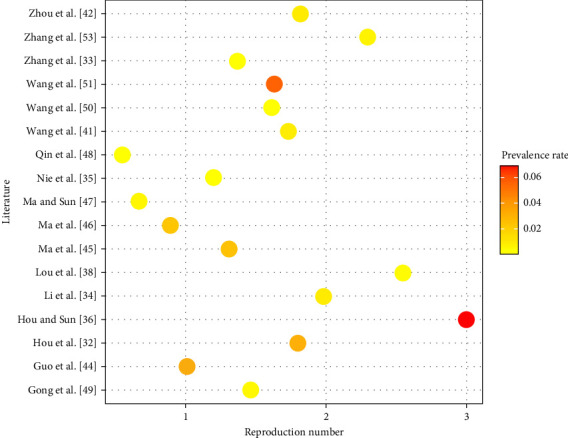
Distribution of the reproduction number and initial livestock individual prevalence rate from the references.

An important indicator used to characterize the prevalence of disease is the reproduction number including basic reproduction number, control reproduction number, and effective reproduction number. In terms of the related parameter values, reproduction number can be calculated.  Figure 4 lists the values of reproduction number as well as the initial animal individual prevalence rate of some regions from the reference. It can be seen that the initial individual positive rate is between 0.02% and 6.9%, and the reproduction number is between 0.55 and 3. The relationship between the initial individual prevalence rate and reproduction number is nonlinear. The data information plays an important role in determining the values of the model parameters. However, when the data information is insufficient, it is very important to construct the structure of the model depending on the epidemiology of Brucellosis (see Section 3.2.2). Therefore, both epidemiology and data are very important in the researches.

### 3.2. Modeling Approaches

#### 3.2.1. Modeling Tool

**Figure 5:**
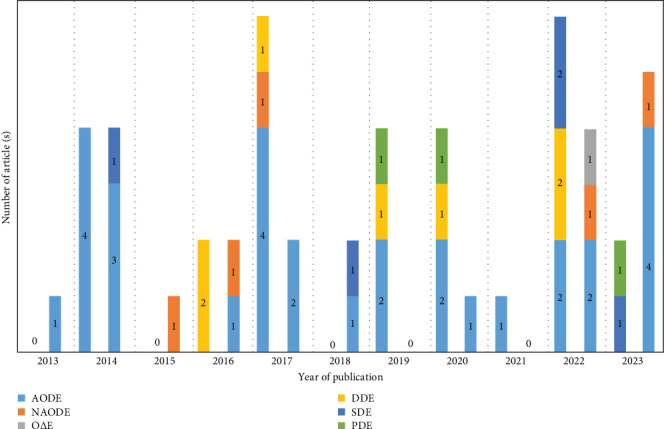
Temporal distribution of the number of published articles and modeling tools on dynamical modeling studies. There are two bars in each year, where the left denotes the theoretical studies and the right represents the applied studies. Define autonomous ordinary differential equation as AODE, nonautonomous, ordinary differential equation as NAODE, ordinary difference equation as OΔE, delay differential equation as DDE, stochastic differential equation as SDE, and partial differential equation as PDE. Notice: Some studies may contain multiple modeling tools.

There are some mathematical tools for dynamic modeling including the differential equation and difference equation, where differential equations include ordinary differential equation (ODE), delay differential equation (DDE), stochastic differential equation (SDE), and partial differential equation (PDE) (see Figure 5). The difference between the two types of equations is whether the time scale is continuous or discrete. In real situations, discrete time scales are more realistic, while the corresponding difference equations are difficult to analyze. That is the reason that most studies focus on continuous time scale and use the ODE model (see Table 1). There is only one study considering the dynamic model governed by difference equations [45]. Usually, difference equations and differential equations can also be transformed into each other, but the property of the solution may vary considerably. The other three types of models (DDE, SDE, and PDE) focus more on the mathematical analyses and lack the integration with actual data (see Table S1).

#### 3.2.2. Modeling Structure

Most of the modeling ideas are derived from the compartmental models, which describe the transmission of diseases and contain a sequence of original effort by Kermack and McKendrick [60–62]. The total population is divided into some subpopulations according to the characteristics of disease transmission. The basic technical route of dynamic modeling is reviewed systematically by Sun et al. [8]. The models focus on population level, considering animal population, human population, and brucella in the environment. Some typical compartmental structures for animal population include susceptible–infectious (SI), susceptible–exposed–infectious (SEI), and susceptible–exposed–infectious–vaccinated (SEIV). Remarkably, the exposed compartment is also infectious in some models, which is confused with classic SEIR models (see Tables S1 and S2). In order to better distinguish, there are also studies with the structures susceptible–recessive infected–quarantined seropositive infected–vaccinated (SRQV) and susceptible–asymptomatic infected–quarantined infected–vaccinated (SAQV). More complex model structures involve multiple groups for animal population such as basic ewe population–other sheep population, cattle population–sheep population, young sheep–adult sheep, and so on. Considering the transportation of animals, the multipatch models have also been taken into account. The division of ewes and other sheep is reasonable since there are evidences that infected pregnant ewes are more infectious. However, attention needs to be paid to the type of farm, for example, there are no ewes for reproduction in the all-in all-out farm, so there is no need to distinguish. Young sheep (lambs) and adult sheep are also worth distinguishing, as there are evidences that the lambs are not contagious and are usually not vaccinated. For human population, the usual structure is susceptible-acute infected-chronic (SAC) infected and also multigroup, that is, high-risk people and low-risk people [42].

#### 3.2.3. Description of Disease Transmission

The transmission of brucellosis usually consists of two aspects: direct transmission and indirect transmission. Direct transmission includes respiratory transmission, exposure to the abortions of infected animals, etc. Indirect transmission is mainly through exposure to bacteria in the environment. There is a big difference between those two transmission modes in the actual situation. For the direct transmission, the susceptible animals are infected with a probability each time they come into contact with an infectious animal. It is worth noting that the probability is different in different cases, and hence it is difficult to obtain its value which is usually estimated by actual data. Indirect transmission means that the susceptible animals are infected when they are exposed to the environment with a certain number of brucella. Transmission is possible only when the number of bacteria in the environment accumulates to a certain extent [63].

Using mathematical formulations to depict the disease transmission process requires referring to some contents in textbooks [26, 64]. For dynamic model, the disease transmission process can be characterized by the so-called incidence rate, *β*_0_*U*(*N*)*I*(*t*)*S*(*t*)/*N*(*t*), which represents the number of individuals who become infected per unit of time at time *t*, where *N* is the total population, *β*_0_ is the transmission probability of each contact, *U*(*N*) is the number of contacts an infection has with others, *S*(*t*)/*N*(*t*) is the proportion of susceptible population in the total population at time *t*, and *I*(*t*) is the infectious population or pathogen at time *t*. The incidence rate usually takes the following forms: mass action incidence rate, standard incidence rate, saturation incidence rate, and so on. Mass action incidence rate is derived by assuming that *U*(*N*) is proportional to *N*, that is, *U*(*N*)=*cN* (*c* is a coefficient), while standard incidence rate is obtained if *U*(*N*) is assumed to be a constant. Different contact patterns can lead to different incidence rates. Based on the characteristics of indirect transmission of brucella, a switching incidence rate is formulated to investigate the scenario that the infection occurs only when the number of bacteria meets or exceeds some certain threshold. Yue, Mu, and Yu [52] carefully compared three common environmental exposure functions used in the literature. It is shown that the threshold dynamics of the system with three functions are similar (see [65]), while the numerical simulations are considerably different.

#### 3.2.4. Characterization of Control Measures

The effectiveness of prevention and control measures is often assessed in these studies. A key question is how to depict these measures in the model. There are usually two main ways of dynamic modeling, one is to parameterize the prevention and control measures in the dynamic model, and the other is to refer the populations with prevention and control measures as state variables. Generally speaking, culling, slaughter, and disinfection measures are described in the first way. Vaccination measures are depicted in the second way. Those parameterized measures are described by a “rate,” which represents the average time some measure is taken, for example, if testing is performed on a herd and the measure is assumed to be implemented in 7 days, then the rate is 1/7 day^−1^. Usually, to isolate a population with some measures as a new variable is because of the heterogeneity between this population and the rest. For instance, the susceptibility of the vaccinated susceptible population to infection will be greatly reduced, thus the vaccinated susceptible population and the nonvaccinated susceptible population should be distinguished as two distinct compartments.

##### 3.2.4.1. Disinfection/Sterilization

Some studies show that the brucellosis in the environment plays an important role in the transmission of the disease (see Table 1). Disinfection or sterilization is a useful measure to eliminate the bacteria in the environment. The disinfection measure is described by disinfection times per unit time and effective disinfection rate each time [32–35]. The disinfection times are suggested as twice a week in Zhejiang [33], where captive breeding is usually the adopted production mode. Li et al. [39] showed a high heterogeneity of threshold disinfection frequency such that the disease can be controlled (i.e., *R*_0_ < 1) among 11 provinces. The effectiveness of disinfection each time cannot be treated as a constant but varies in a range from 0.15 to 0.9. The difference is closely associated with the operation of the related personnel. It has been demonstrated that the disinfection cannot eliminate the disease but is statistically significant with the magnitude of the disease among livestock [32, 35, 45] and humans [43] in Jilin, Inner Mongolia, where those pastoral regions mainly depend on grazing breeding but also include other production modes such as captive breeding, mixed breeding, etc. In fact, for pastoral areas without pens, disinfection measures are almost impossible since it is very difficult to implement this measure on the vast grassland. For studies related to pastoral areas, it is noteworthy that, due to the heterogeneity in production modes, the rather tricky issue is how to combine the research results on this measure with the actual situation.

##### 3.2.4.2. Vaccination

Vaccines can be used to provide the immunization protection to susceptible population. The success stories of eradicating brucellosis in many countries show that the vaccination is carried out when the prevalence rate of the disease among livestock is high (usually larger than 1% or 2%). Vaccination is considered not only an effective but also a cost-effectiveness measure in reality [40, 50]. In the model, it is described by both the variable (i.e., the number of vaccinated individuals) and the parameters, such as vaccination rate, losing immunity rate, and effectiveness of vaccination. Here, the reciprocal of vaccination rate means the average waiting time from susceptible population to vaccinated population. On the contrary, the reciprocal of losing immunity rate denotes the average waiting time from vaccinated population to susceptible population, that is, the average protection period for vaccines. The effectiveness of vaccination is usually related to the vaccine itself. Because the process of vaccination also involves professional people and the real vaccine coverage rate admits a difference in terms of people's operations.

Some models assume that the vaccinated individuals cannot be infected [34, 42, 44, 45, 49], while other models suppose that the vaccinated individuals may still be infected but are less likely to be infected than those not be vaccinated [32, 36, 38]. Regardless of the modeling approach, the conclusion is that vaccination alone cannot eradicate the disease [38, 43, 45]. In terms of the study in Inner Mongolia, if the immunization rate remains at the estimated value from 2016 to 2020, the number of new infections will fluctuate around from 2000 to 5000 (see Figure 6c in [45]). Further, even in the case of a very high immunization rate, new infections still exist and range around 400–1400 (see Figure 11 in [45]). Peng et al. [43] simulate that, when the vaccine coverage changes from 0% to 80%, acute human brucellosis incidence would decrease from 0.2% to under 0.05%. In addition to explore the implementation intensity of vaccine coverage, they also compare some scenarios with vaccine types in nationwide, which are S2 vaccine in sheep and cattle, RB51 vaccine in cattle and Rev-1 vaccine in sheep, S2 vaccine in cattle and sheep, and RB51 in cattle and Rev-1 vaccine in sheep (see Figure 3 in [43]). They conclude that S2 vaccine compared with RB51 and Rev-1 vaccine could lead to less human brucellosis incidence.

##### 3.2.4.3. Testing–Slaughter/Detection/Culling/Elimination

Testing and slaughter are two different measures. In some studies, the slaughter is also called as culling or elimination and the testing is called as detection. Since they are usually implemented together, testing and slaughter are considered as one key measure to control the animal disease. Sensitivity analysis on reproduction number shows that testing–slaughter is statistically more significant than other measures [34, 35, 42, 50]. Some studies show that testing–slaughter is also extremely effective in reducing the infections among humans and livestock [32, 38, 52].

The studies create some quantitative results in different regions to achieve the objectives required by the governments [45, 49]. In the study of Ningxia, the measure seems to have little effect to control brucellosis in humans. The authors indicate that merely increasing the slaughter rate of sick sheep cannot enable to achieve the goal of local authority in 2024 either, while the rate should increase by 30% on the premise of maintaining the improvement of other measures to achieve the goal (see Figure 5 in [49]). Studies on Jilin show that if the culling rate is increased by 20%, the goal (i.e., human brucellosis cases fall below 240) will be achieved 10 years ahead of schedule (see Figure 5 in [46]). In reality, due to the large number of livestock and the limited human resources, the testing coverage is not 100%. Even if the 100% test rate is achieved, the sensitivity and specificity of detection reagent may still lead to false positive or false negative.

Under different situations and goals, the effectiveness of the measure is not consistent. Another issue that needs to be considered is that culling measure is not a cost-effective way to control the disease. The high slaughter rate of infected livestock can generate the most economic damage to the herdsman. Therefore, in practice, this measure is implemented in situations with a low prevalence rate and needs to be evaluated in combination with the local fiscal revenue. An interesting numerical simulation in the case of Jilin by Zhang et al. [40] exhibits that the entire flock detection does not cost the most, while the detection cost is maximized when the detection rate is 0.4.

##### 3.2.4.4. Import/Transport Restriction

There are many production systems including self-supplying farms, all-in all-out farms, and so on. No matter the system, the movement of livestock is indispensable. Few quantitative studies have explored the impact of the measures on livestock movement such as import restriction and transport restriction. Existing studies usually explore the effect of transportation by establishing patch model, which is the model that divides a space into discrete patches and analyzes the interactions and dynamics within and between these patches. Model simulations show that the transport patterns have a great impact on disease transmission [48]. A study from Guo et al. [44] shows that even though the basic reproduction number for each region is less than one, the basic reproduction number of whole regions is larger than one when the transportation occurs, so the disease is still persistent. If the transportation route is two-way between two regions or one-way from high-risk to low-risk region, then the reproduction number will increase with the increasing transport rate. When the route is only from low-risk region to high-risk region, the reproduction number first decreases then increases with the increase of transport rate [44]. A case study in Zhejiang suggests to insist on self-supplying production of the dairy cows when the importation of infected animals cannot be avoided. Nie et al. [35] pointed out that the effect of prevention and control measures is closely related to the amount of dairy stocking and importing of cattle.

##### 3.2.4.5. Cutting Mixed Cross-Infection/Isolation/Quarantine

In terms of disease prevention and control, dynamical models are capable of depicting the comprehensive impact of measures such as isolation, quarantine, and cutting mixed cross-infection [34, 66]. In the prevention of disease, this measure is reflected by raising different ages animals, raising different species (e.g., sheep and cow), and raising animals transported at different frequencies in separate groups. In particular, abortion and pregnant animals have the potential risk of the transmission of the disease [67]. With regard to controlling the development of the disease, the measure includes separating infected animals from healthy ones through isolation and quarantine methods. Specifically, animals suspected of being infected with brucellosis are promptly isolated and monitored. Areas where infected animals are found are quarantined to restrict the movement of animals.

The modeling study on Inner Mongolia shows that the reproduction number decreases from 1.3167 to 0.9868 if mixed cross-infection between basic ewes and other sheep is cutoff with the same vaccination rate and the seropositive detection rate [34]. If there exists the bidirectional nature of mixed cross-infection between cattle and sheep, the disease cannot be extinct even if the reproduction number of brucellosis in sheep flocks and cattle herds is less than 1 [66]. In addition, isolation or quarantine of the suspected and infected animals is very crucial when the animals are imported [33, 43, 68].

## 4. Discussion

Dynamic models contribute qualitative and quantitative findings and play a vital role in the study of epidemiological dynamics of brucellosis. From the preceding studies mentioned above, one has a deep understanding of the application of dynamic model in the study of brucellosis in China, in particular, the explorations of prevention and control measures. The purpose of this study is to summarize the past experiences, find the limitations of modeling study, clarify the key scientific issues, and lay a solid foundation for the future research. There are still some challenging problems in dynamical modeling that need to be explored, which are listed in the following.

### 4.1. Selection of Model Structure

For a new modeler, the first thing that should be seriously and carefully thought about is model selection. Usually, the SVEIR and SEIR models are adopted by most scholars to expound the transmission of brucellosis with and without vaccination. By reviewing the literature, some researchers assume that “E” is infectious [33, 34], while others assume that “E” is not infectious [43, 69]. Similarly, for the vaccinated population “V,” some models assume that “V” cannot be infected [70], and others suppose that it can be infected by a lower force of infection [32]. Therefore, the structure of the models is still confusing, that is, (1) Is “E” (exposed population) in the model infectious? (2) and Can “V” (vaccinated population) be infected? For the multigroup model proposed by [65], it has been theoretically proved that the threshold dynamics can be achieved whether or not the infectious “E” is incorporated into the models, and the model with “E” has a larger reproduction number. In fact, the infectious “E” can be considered asymptomatic infection, while “E” which is not infectious can be considered exposed phase, and the infectious role reflects on “I.” For the vaccinated population “V,” it is difficult to make “V” completely immune in reality. Therefore, it is reasonable that “V” can be infected. Otherwise, it is reasonable that “V” is not infected if the accurate vaccine protective rate is obtained.

Another key mathematical parameter determining the incidence rate is the transmission probability. Most models are deterministic with continuous time, so this probability is also a constant. While it is quite realistic to consider the stochastic epidemic process in discrete time. Chain binomial models assume that the transmission probability is binomial (see [71, 72] for more details), which were first developed by Reed and Frost in 1928 ([29, 73–75], reference therein). It can be considered that the susceptible is infected or not after each contact as an event. Then, the transmission process can be seen as a binomial distribution. Discrete and stochastic model is more realistic, but is theoretically more complex to analyze.

### 4.2. Usage of Data

The quantitative results cannot be well characterized without the use of data. It is important to know what data we have. The type of data determines the structure of the model, which is called data-driven modeling. The primary purpose of data in dynamic models is used for calibration and validation. Some parameters can be calculated directly from data information, while those related to force of infection are usually estimated using infection data or testing positive data. The latter is to fit the model output with existing data information. However, testing methods and immunization background are rarely mentioned in the existing studies. A better understanding of the type and validity of tests and vaccines will facilitate an effective application of the model. Nevertheless, different immunization backgrounds and testing methods are, respectively, described by the same parameters in the model but with differences in values.

Typically, the data types used in the dynamic model of brucellosis include human-confirmed cases and the number of testing positive of livestock. Indeed, livestock-related data are more useful for understanding disease infection in livestock, but are difficult to obtain in reality. For zoonoses, it provides another way to get unknown parameters (e.g., force of infection among livestock and from livestock to human) from the model based on data of human infections. That is just the way that most of the researchers used to do based on a review of the literature (see Table 1). However, the lack of further model validation makes the reliability of the model results doubtful or questionable. A way to validate is via cross-validation by multisource data types, for example, two sets of data are applied, one is for estimating parameters, and the other is for testing.

### 4.3. Limitations of Current Dynamic Models

#### 4.3.1. Lack of Heterogeneity

Existing researches lack the heterogeneity. The heterogeneity is due to the complexity and diversity of the reality. In fact, “heterogeneity” involves many aspects. For example, the heterogeneity of breeding scale, such as backyard, large-scale farms, and so on; the heterogeneity of production system, that is, self-breeding farms, all in-all out farms, and so on; the heterogeneity of farming patterns, captive farming, seasonal grazing, captive grazing, etc. However, not all the heterogeneity needs to be considered, and sufficient knowledge of risk factors is necessary to further explore which heterogeneity needs to be incorporated into the model.

It is not difficult to observe that the contact pattern is one of the important links in portraying the transmission of disease. Contact patterns provide a prerequisite for the transmission of disease. For livestock diseases, the contact pattern of animals varies at different levels. A simple way to describe the contact pattern of each animal is provided by an average constant contact number assuming that the population is homogeneous. However, for captive production systems, the animals in different farming units do not contact with general situation. In addition, some measures, especially quarantines, also prevent isolated animals from coming into contact with nonisolated animals. Similarly, the contact pattern of humans and animals is determined by many factors, for example, the occupational people have more contact with animals than other people.

#### 4.3.2. Lack of Studies on Herd/Flock Level

In the field of veterinary epidemiology, there are some key indicators to describe the development of disease such as incidence rate, prevalence rate, and so on. From different scales, these indicators can be divided into individual-level indicator and herd/flock-level indicator. It is worth mentioning that the indicators related to herd/flock level are useful for understanding the distribution of diseases in a given region, which is essential for making decisions.

The results obtained by the existing models are all at the individual level. This is because the current models are established in terms of the changes in the number of individuals but not the number of herds/flocks. Therefore, the key to get information on herd/flock level is to build a model based on the variation in the number of herds/flocks. This requires some investigation of the actual situation, such as the pattern of contact between each herd/flock, that is, the social networks of those herds/flocks. In fact, the information of this kind is difficult to obtain in a large and complex farming region. When there is insufficient information, the charm of dynamic models is that different scenarios can be well simulated through “what-ifs.”

#### 4.3.3. Lack of Quantitative Characterization of Relation Between Human and Livestock Cases

From Table S1, it is observed that the main quantitative findings of dynamic models focus on the analysis of threshold dynamics. In reality, human incidence is more likely to detect than that of livestock. It is very natural and reasonable to ask whether it is possible to determine the prevalence of infection in livestock from that in human. However, the quantitative relationship between human and livestock infection is still unclear, and almost all the existing models ignore this point while focus on only one aspect, that is, the variation of the number of infection cases in human or in livestock.

Zhou et al. [42] formulated a multigroup model to investigate brucellosis transmission among sheep and two human subpopulations (i.e., high-risk and low-risk) and to explore the prevalent dynamics of brucellosis in Inner Mongolia from 2001 to 2011. It is found that the variations of both human incidence and livestock incidence are very sensitive to the intensity of prevention and control measures. However, the quantitative relationship between the incidences in sheep and human is not explicitly well established. It is suggested that such relationship can be well characterized by mathematical–dynamical modeling approach by considering the actual situation although it seems very challenging.

## 5. Conclusions and Prospects

Brucellosis is a zoonotic infection transmitted from infected animals to humans and is common in animal husbandry. Recently, the number of human brucellosis cases has reached a new high record, which reflects that the situation of livestock brucellosis cases is also not optimistic. Dynamic modeling as a useful tool provides valuable insights into the prevention and control of the disease. Modelers in China use this method to study the prevent and control of brucellosis since 2013. This article systematically summarizes the application of dynamic model to brucellosis in China, illustrates the gap between theory and practice, and provides some instructive and illuminating perspectives for future research. However, modeling studies are not limited to dynamic models alone but other types of modeling studies are less mentioned here. In addition, modeling studies on brucellosis in other countries have been less well presented in this review article. By reviewing the existing publications, less modeling studies investigate the prevention and control of brucellosis driven by animal data. In fact, a vital motivation for introducing the dynamic model lies in the lack of data. Even with less data and the cognition of the characteristics of disease transmission, the dynamic model can still be established. Thereafter, by carrying out theoretical analysis and numerical simulation of the model, the dynamical evolution of the disease under different scenarios can be well explored. It has been demonstrated that dynamic modeling studies are different from the studies that require a large amount of data for statistical analysis and serve as some useful references for practice. Of course, an appropriate model combined with accurate data will provide better understanding of and deeper insight into the prevention and control.

As mentioned in Section 4 (Discussion section), future researches should also pay more attention to heterogeneity and herd/flock level outcomes. As can be seen from the recent studies, researchers do not just consider the characteristics of disease transmission in models. They also examine the effect of human behavior, ecological environment, social factors, and so on. Recent findings highlight that the socioeconomic factors play a more significant role in the spread of human brucellosis than other factors [20]. Those are in line with the One Health approach, which is applicable to the zoonoses. However, from the information in Table 1, it is evident that there is limited research involving human, economic, and environment factors. As the number of factors under consideration increases, it is natural that the analysis of models becomes more complex. This underscores the need for a deeper integration with computer techniques to improve computation and yield simulation results. In the future, close collaboration among different departments and disciplines must be strengthened.

## Figures and Tables

**Table 1:** Control measures and factors considered in the literature.

Literature	Host type	Measures					Factors		
		CM-1	CM-2	CM-3	CM-4	CM-5	Human	Economic	Environment
[32]	Sheep–human	√	√	√	—	—	—	—	—
[33]	Dairy cattle	√	—	√	√	—	—	—	—
[34]	Sheep–human	√	√	√	—	√	—	—	—
[35]	Dairy cattle	√	—	√	√	—	—	—	—
[36]	Sheep	—	√	√	—	—	—	—	—
[37]	Sheep–human	—	—	—	—	—	—	—	—
[38]	Sheep/cattle–human	—	√	√	—	—	—	—	—
[39]	Sheep–human	√	√	√	—	—	—	—	—
[40]	Sheep–human	—	√	√	—	—	—	√	—
[41]	Sheep/cattle–human	—	—	—	—	—	—	—	—
[42]	Sheep–human	—	√	√	—	—	√	√	—
[43]	Sheep–cattle–human	√	√	—	—	√	√	—	—
[44]	Sheep–human	—	√	√	√	—	—	—	—
[45]	Sheep–human	—	√	√	—	√	√	—	—
[46]	Sheep–human	√	—	√	—	—	√	—	—
[47]	Sheep–human	√	√	√	—	—	—	—	√
[48]	Sheep–human	—	—	√	√	—	—	—	—
[49]	Sheep–human	—	—	√	—	—	√	—	—
[50]	Livestock	√	—	√	—	—	—	√	—
[51]	Sheep–human	√	—	√	—	√	—	√	—
[52]	Sheep	√	—	√	—	—	—	—	—
[53]	Sheep–human	—	—	—	—	—	—	—	√

*Note*: Different articles on the same control measure are not uniform, so we unify this. Control measure-1 (CM-1): disinfection/sterilization; control measure-2 (CM-2): vaccination; control measure-3 (CM-3): testing and slaughter/detection/culling/elimination; control measure-4 (CM-4): import/transport restriction; control measure-5 (CM-5): cutting mixed cross infection/isolation/quarantine.

Abbreviations: CM-1, control measure-1; CM-2, control measure-2; CM-3, control measure-3; CM-4, control measure-4; CM-5, control measure-5.

## Data Availability

The data that support the findings of this study are available within the manuscript and its supporting information files.

## Supporting Information

Supporting Information Table S1: Overview of the articles on theoretical researches included in this review. Table S2: Overview of the articles on applied researches included in this review.

## References

[1] Corbel M. J. (2006). *Brucellosis in Humans and Animals*.

[2] Abernethy D. A., Moscard-Costello J., Dickson E. (2011). Epidemiology and Management of a Bovine Brucellosis Cluster in Northern Ireland. *PreventiveVeterinary*.

[3] Benkirane A. (2006). Ovine and Caprine Brucellosis: World Distribution and Control/Eradication Strategies in West Asia/North Africa Region. *Small Ruminant Research*.

[4] Davidson R. M. (2002). Control and Eradication of Animal Diseases in New Zealand. *Zealand Veterinary Journal*.

[5] Peng C., Li Y. J., Huang D. S., Guan P. (2020). Spatial-Temporal Distribution of Human Brucellosis in Mainland China from 2004 to 2017 and an Analysis of Social and Environmental Factors. *Environmental Health and Preventive Medicine*.

[6] Dadar M., Tiwari R., Sharun K., Dhama K. (2021). Importance of Brucellosis Control Programs of Livestock on the Improvement of One. *Veterinary Quarterly*.

[7] NDCPA (2023). National Disease Control and Prevention Administration. https://www.ndcpa.gov.cn/jbkzzx/c100016/common/list.html.

[8] Sun G. Q., Li M. T., Zhang J., Zhang W., Pei X., Jin Z. (2020). Transmission Dynamics of Brucellosis: Mathematical Modelling and Applications in China. *Computational and Structural Biotechnology Journal*.

[9] Lai S., Chen Q., Li Z. (2021). Human Brucellosis: An Ongoing Global Health Challenge. *China CDC Weekly*.

[10] CAHEC (2021). China Animal Health and Epidemiology Center Circular of the Office of the Ministry of Agriculture and Rural Affairs on the Promotion of Construction and Evaluation of Epidemic-Free and Epidemic-Free Areas for Animals Such as Cattle and Sheep Disease (Agriculture and Pastoralism [2021] no. 32). https://www.cahec.cn/detail/42828.html.

[11] Zhang N., Huang D., Wu W. (2018). Animal Brucellosis Control or Eradication Programs Worldwide: A Systematic Review of Experiences and Lessons Learned. *Preventive Veterinary Medicine*.

[12] MARAPR (2021). Ministry Agriculture and Rural Affairs of the People’s Republic of China. Notice of the National Plan for Compulsory Immunization Against Animal Diseases 2. http://www.moa.gov.cn/govpublic/xmsyj/202101/t20210111_6359742.htm.

[13] Keeling M. J., Woolhouse M. E., Shaw D. J. (2001). Dynamics of the 2001 UK Foot and Mouth Epidemic: Stochastic Dispersal in a Heterogeneous Landscape. *Science*.

[14] Keeling M. J., Woolhouse M. E. J., May R. M., Davies G., Grenfell B. T. (2003). Modelling Vaccination Strategies Against Foot-and-Mouth Disease. *Nature*.

[15] Hayes B. H., Andraud M., Salazar L. G., Rose N., Vergne T. (2021). Mechanistic Modelling of African Swine Fever: A Systematic Review. *Preventive Veterinary Medicine*.

[16] Lambert S., Bauzile B., Mugnier A., Durand B., Vergne T., Paul M. C. (2023). A Systematic Review of Mechanistic Models Used to Study Avian Influenza Virus Transmission and Control. *Veterinary Research*.

[17] Alim M., Ye G. H., Guan P., Huang D. S., Zhou B. S., Wu W. (2020). Comparison of ARIMA Model and XGBoost Model for Prediction of Human Brucellosis in Mainland China: A Time-Series. *BMJ Open*.

[18] Wang Y., Xu C., Zhang S., Wang Z., Zhu Y., Yuan J. (2018). Temporal Trends Analysis of Human Brucellosis Incidence in Mainland China From 2004 to 2018. *Scientific Reports*.

[19] Zheng Y., Zhang L., Wang C. (2021). Predictive Analysis of the Number of Human Brucellosis Cases in Xinjiang. *Scientific Reports*.

[20] Wen X., Wang Y., Shao Z. (2024). The Spatiotemporal Trend of Human Brucellosis in China and Driving Factors Using Interpretability. *Scientific Reports*.

[21] Bie S. Y., Zhang H. G. (2022). Analysis of the Distribution Characteristics and Influencing Factors of Brucellosis in China Based on Spatial-Temporal Weighted Poisson Regression Model. *China Journal of Health Statistics*.

[22] Havas K. A., Boone R. B., Hill A. E., Salman M. D. (2014). A Brucellosis Disease Control Strategy for the Kakheti Region of the Country of Georgia: An Agent-Based Model. *Zoonoses and Public Health*.

[23] Zhai M., Li W., Tie P. (2021). Research on the Predictive Effect of a Combined Model of ARIMA and Neural Networks on Human Brucellosis in Shanxi Province: A Time Series Predictive Analysis. *BMC Infectious Diseases*.

[24] Gillman M. (2005). Population dynamics: introduction. *eLS*.

[25] Zhao X., Liu L., Wang H., Fan M. (2023). Ecological Effects of Predator Harvesting and Environmental Noises on Oceanic Coral Reefs. *Bulletin of Mathematical Biology*.

[26] Brauer F., Castillo-Chavez C., Feng Z. (2019). *Mathematical Models in Epidemiology*.

[27] Carpenter T. E., Berry S. L., Glenn J. S. (1987). Economics of *Brucella ovis* Control in Sheep: Epidemiologic Simulation Model. *Journal of the American Veterinary Medical*.

[28] Dobson A., Meagher M. (1996). The Population Dynamics of Brucellosis in the Yellowstone National Park. *Ecology*.

[29] Hurd H. S., Kaneene J. B. (1993). The Application of Simulation Models and Systems Analysis in Epidemiology: A Review. *Preventive Veterinary Medicine*.

[30] Zinsstag J., Roth F., Orkhon D. (2005). A Model of Animal-Human Brucellosis Transmission in Mongolia. *Preventive Veterinary Medicine*.

[31] Liberati A., Altman D. G., Tetzlaff J. (2009). The PRISMA Statement for Reporting Systematic Reviews and Meta-Analyses of Studies that Evaluate Health Care Interventions: Explanation and Elaboration.. *Annals of Internal Medicine*.

[32] Hou Q., Sun X., Zhang J., Liu Y., Wang Y., Jin Z. (2013). Modeling the Transmission Dynamics of Sheep Brucellosis in Inner Mongolia Autonomous Region, China. *Mathematical Biosciences*.

[33] Zhang J., Sun G. Q., Sun X. D. (2014). Prediction and Control of Brucellosis Transmission of Dairy Cattle in Zhejiang Province, China. *Plos One*.

[34] Li M., Sun G., Zhang J. (2014). Transmission Dynamics and Control for a Brucellosis Model in Hinggan League of Inner Mongolia, China. *Mathematical Biosciences and Engineering*.

[35] Nie J., Sun G. Q., Sun X. D. (2014). Modeling the Transmission Dynamics of Dairy Cattle Brucellosis in Jilin Province, China. *Journal of Biological Systems*.

[36] Hou Q., Sun X. D. (2015). Modeling Sheep Brucellosis Transmission With a Multi-Stage Model in Changling County of Jilin Province, China. *Journal of Applied Mathematics and Computing*.

[37] Zhang J., Jin Z. (2015). The Application of the Nonautonomous Dynamics Model on Brucellosis in Hinggan League(in Chinese). *Journal of Inner Mongolia Normal University*.

[38] Lou P., Wang L., Zhang X., Xu J., Wang K. (2016). Modelling Seasonal Brucellosis Epidemics in Bayingolin Mongol Autonomous Prefecture of Xinjiang, China, 2010-2014. *BioMed Research International*.

[39] Li M. T., Sun G. Q., Zhang W. Y., Jin Z. (2017). Model-Based Evaluation of Strategies to Control Brucellosis in China. *Journal of Environmental Research and Public*.

[40] Zhang J., Jin Z., Li L., Sun X.-D. (2017). Cost Assessment of Control Measure for Brucellosis in Jilin Province, China. *Chaos, Solitons & Fractals*.

[41] Wang L., Wang K., Jiang D., Hayat T. (2018). Nontrivial Periodic Solution for a Stochastic Brucellosis Model With Application to Xinjiang, China. *Physica A: Statistical Mechanics and Its Applications*.

[42] Zhou L., Fan M., Hou Q., Jin Z., Sun X. (2018). Transmission Dynamics and Optimal Control of Brucellosis in Inner Mongolia of China. *Mathematical Biosciences and Engineering*.

[43] Peng C., Zhou H., Guan P., Wu W., Huang D. S. (2020). An Estimate of the Incidence and Quantitative Risk Assessment of Human Brucellosis in Mainland China. *Transboundary and Emerging Diseases*.

[44] Guo J., Luo X., Zhang J., Li M. (2022). A Mathematical Model for Ovine Brucellosis during Dynamic Transportation of Sheep, and Its Applications in Jalaid Banner and Ulanhot City. *Mathematics*.

[45] Ma X., Li M., Zhang J., Luo X., Sun G.-Q. (2022). Interactions of Periodic Birth and Shearing Induce Outbreak of Brucellosis in Inner Mongolia. *International Journal of Biomathematics*.

[46] Ma X., Sun G. Q., Wang Z. H., Chu Y. M., Jin Z., Li B. L. (2022). Transmission Dynamics of Brucellosis in Jilin Province: Effects of Different Control Measures. *Communications in Nonlinear Science and Numerical Simulation*.

[47] Ma X., Sun G. Q. (2024). Global Dynamics of a Periodic Brucellosis Model With Time Delay and Environmental Factors. *Applied Mathematical Modelling*.

[48] Qin Y., Pei X., Li M., Chai Y. (2022). Transmission Dynamics of Brucellosis With Patch Model: Shanxi and Hebei Provinces as Cases. *Mathematical Biosciences and Engineering*.

[49] Gong W., Sun P., Zhai C. (2023). Accessibility of the 3-Year Comprehensive Prevention and Control of Brucellosis in Ningxia: A Mathematical Modeling Study. *BMC Infectious Diseases*.

[50] Wang L. S., Li M. T., Pei X., Zhang J., Sun G. Q., Jin Z. (2023). Cost Assessment of Optimal Control Strategy for Brucellosis Dynamic Model Based on Economic Factors. *Communications in Nonlinear Science and Numerical Simulation*.

[51] Wang Y., Cai Y., Li M., Liu J. (2023). Modeling of Brucellosis Dynamics and Its Control Measures Based on Price Factors. *Advances in Applied Mathematics*.

[52] Yue Z., Mu Y., Yu K. (2023). Dynamic Analysis of Sheep Brucellosis Model With Environmental Infection Pathways. *Mathematical Biosciences and Engineering*.

[53] Zhang Z., Ma X., Zhang Y., Sun G., Zhang Z. K. (2023). Identifying Critical Driving Factors for Human Brucellosis in Inner Mongolia, China. *Physica A: Statistical Mechanics and Its Applications*.

[54] PHSD The Data-Center of China Public Health Science. *National Population Ans Health Science Data Sharing Platform*.

[55] Cui B. Y., Jiang H. (2018). Surveillance Data of Brucellosis in China, 2005–2016. *Disease Surveillance*.

[56] DTAB (2002). Diagnostic Techniques for Animal Brucellosis. https://openstd.samr.gov.cn/bzgk/gb/std_list?p.p1=0&p.p90=circulation_date&p.p91=desc&p.p2=GB/T%2018646-2002.

[57] DTAB (2018). Diagnostic Techniques for Animal Brucellosis. https://openstd.samr.gov.cn/bzgk/gb/newGbInfo?hcno=1B08331FA7B4B3219281079391BB8BB2.

[58] CDC (2020). Center for Public Health Data Science By Region. https://www.phsciencedata.cn/Share/ky_sjml.jsp?id=aafa8285-42ae-4dbc-a828-152c2cef6396.

[59] Rossetti C. (2023). CABI Compendium.

[60] Kermack W. O., McKendrick A. G. (1927). A Contribution to the Mathematical Theory of Epidemics.. *Proceedings of the Royal Society of London. Series A*.

[61] Kermack W. O., McKendrick A. G. (1932). A Contribution to the Mathematical Theory of Epidemics, Part II-the Problem of Endemicity. *Proceedings of the Royal Society of London Series A*.

[62] Kermack W. O., McKendrick A. G. (1933). A Contribution to the Mathematical Theory of Epidemics, Part III-Further Studies of the Problem of Endemicity.. *Proceedings of the Royal Society of London Series A*.

[63] Yang C., Gao J., Xian R. (2024). Molecular Epidemiology of *Brucella abortus* Isolated From the Environment in Ningxia Hui Autonomous Region, China. *Infection, Genetics and Evolution*.

[64] Kretzschmar M., Wallinga J. (2010). Mathematical Models in Infectious Disease Epidemiology.

[65] Li M. T., Jin Z., Sun G. Q., Zhang J. (2017). Modeling Direct and Indirect Disease Transmission Using Multi-Group Model. *Journal of Mathematical Analysis and Applications*.

[66] Li M. T., Sun G. Q., Wu Y. F., Zhang J., Jin Z. (2014). Transmission Dynamics of a Multi-Group Brucellosis Model with Mixed Cross Infection in Public Farm. *Applied Mathematics and Computation*.

[67] Li L. M., Wang Q., Shi J. F. (2023). Seroprevalence and Potential Risk Factors of Brucellosis in Sheep From America, Africa and Asia Regions: A Systematic Review and Meta-Analysis. *Research in Veterinary Science*.

[68] Zhang J., Ruan S., Sun G., Sun X., Jin Z. (2014). Analysis of a Multi-Patch Dynamical Model about Cattle Brucellosis. *Journal of Shanghai Normal University (Natural Sciences Mathematics)*.

[69] Hou Q., Wang T. (2017). Global Stability and a Comparison of SVEIP and Delayed SVIP Epidemic Models With Indirect Transmission. *Communications in Nonlinear Science and Numerical Simulation*.

[70] Sun G. Q., Zhang Z. K. (2014). Global Stability for a Sheep Brucellosis Model With Immigration.

[71] Bailey N. T. J. (1975). *The Mathematical Theory of Infectious Diseases and Its Applications*.

[72] Daley D. J., Gani J. (1999). *Epidemic Modelling*.

[73] Abbey H. (1952). An Examination of the Reed-Frost Theory of Epidemics.. *Human Biology*.

[74] Jacquez J. A. (1987). A Note on Chain-Binomial Models of Epidemic Spread: What Is Wrong With the Reed-Frost Formulation?. *Mathematical Biosciences*.

[75] Keeling M. J., Rohani P. (2011). *Modeling Infectious Diseases in Humans and Animals*.

