# Supplementary material for: Dynamic Modeling of Prevention and Control of Brucellosis in China: A Systematic Review
Source: Transbound Emerg Dis. 2025 Jan 11;2025:1393722. doi: 10.1155/tbed/1393722 (PMC12017112; doi:10.1155/tbed/1393722)
Supplement: Supporting Information — Table S1: Overview of the articles on theoretical researches included in this review. Table S2: Overview of the articles on applied researches included in this review. [file 1393722.f1.docx]

**Supplementary Materials**

**Table A1: Overview of the articles on theoretical researches included in this review.**

| Literature | Model structure | Main finding |
| --- | --- | --- |
| Hou et al., [2014](#Hou2014) | Cont. ODE; General input w.r.t. susceptible; General incidence rate  General animal(SEI)-Brucella | The uniqueness of the endemic equilibrium, and the global asymptotically stability of the equilibria |
| Li et al., [2014a](#Li2014a) | Cont. ODE; Constant input rate; Mass action incidence rate  Cattle(SEIV)-Sheep(SEIV)- Brucella | Threshold dynamics; Take measures to remove mixed cross infection, i.e., to prohibit mixed feeding |
| Sun et al., [2014](#sun2014) | Cont. ODE; Constant and linear input rate; Mass action incidence rate  Sheep(SEIV)-Brucella | Elimination, vaccination and disinfection are the useful control strategies; To reduce immigration and self-sufficiency of the flock for controlling sheep brucellosis |
| Zhang et al., [2014b](#zhang2014b) | Multi-patch cont. ODE; Constant input rate; Mass action incidence rate; Cattle(SEI)-Brucella | Threshold dynamics; The emigration of the brucella carriers or the diffusion of brucella in patch whose rasing quantity of cattle is larger can increase R_0_ |
| Hou et al., [2016a](#Hou2016a) | Multi-group DDE(time delay: latency of infection) ;General animals(SI)-Brucella | Threshold dynamics; Delay induces bifurcation |
| Hou et al., [2016b](#Hou2016b) | DDE(time delay: latency of infection); Constant input rate; General incidence rate  General animals(SEI)-Brucella | Threshold dynamics; Time delay is harmless for the stability of equilibria |
| Hou et al., [2017](#Hou2017) | ODE and DDE; General animals(SVEI-ODE)(SVI-DDE)-Pathogen | Time delay is harmless for the stability of equilibria of system; The vaccination rate and the incubation period has a significant influence on the spread of animal diseases |
| Li et al., [2017b](#Li2017b) | Cont. ODE;  Basic ewes(SRiQV)-Other sheep(SRiQV)-Human(SAC) | Threshold dynamics |
| Li et al., [2017c](#Li2017c) | Cont. ODE; Multi-group general animals(SEI)- Pathogen | Threshold dynamics |
| Yang et al., [2017](#yang2017) | Two-patch non-autonomous cont. ODE; General animal(SI)-environmental pathogen | Threshold dynamics; Underscore the importance of including spatial and seasonal heterogeneities in the design of control strategies for brucellosis |
| Hou et al., [2019](#Hou2019) | DDE distributed time delay  General animal: young susceptible-adult susceptible-infected compartment  pathogens in the environment | Distributed time delay is harmless for the dynamics of the spread of brucellosis; Periodic phenomena are found by numerical analysis |
| Li et al., [2019](#Li2019) | Cont. ODE; Basic ewes(SRiQV)-Other sheep(SRiQV)-brucella-  Human(SAC) | Threshold dynamics; Vaccination rate of sheep and seropositive detection rate of recessive infected sheep are very important factor for brucellosis |
| Yang et al., [2019](#yang2019) | PDE &DDE, Animal(SEI)-brucella | Threshold dynamics; The herdsmen should make efforts to keep the living environment of their animals clean, and eliminate infectious animals timely. |
| Zhang et al., [2019](#zhang2019) | Switch ODE; a switch incidence term w.r.t environmental brucellosis (minimum infection dose)  Dairy cows(SEI) | Different amounts of R_0_ may lead to different steady states of brucellosis, and considering the effects of immunology is more serious in mathematics and biology |
| Hou et al., [2020](#Hou2020) | DDE (the time delay in gathering information)  Animal(SEI)-Brucellosis | Considering testing–culling strategy; Test rate had a significant effect on the scope of periodic oscillation, and even it can cause periodic oscillation to disappear |
| Huang et al., [2020](#Huang) | Same with Li et al., [2019](#Li2019) | Exploring the precontrol (the amount of glove-wearing was evident and significant ) |
| Liang et al., [2020](#Liang2020) | ODE with switched transport rate  susceptible-infected | Transport has dual effects;Transport can lead to increase in the number of infected animals; Transport can also reduce the number of infected animals in a certain range; The number of infected animals can be controlled if animals are transported reasonably. |
| Yang et al., [2020](#yang2020) | PDE with non-local diffusion; Spatial and temporal heterogeneity  Animals: susceptible-infectious  Brucella | Time heterogeneity enhances magnitudes of oscillations of animal population and brucella; Enlarging effective infection radii increases the risk of brucellosis propagations; Herdsmen should iprove the sanitation of animals’ environment and isolate infected animals instantly to control brucellosis prevalence |
| Nie et al., [2021](#nie2021) | ODE with pulse control | The existence and orbital asymptotical stability of positive order-1 or order-2 periodic solution; The proportion of infected class can be controlled at a desired low level for a long time and disease will not break out among population. |
| Dang et al., [2022](#Dang) | SDE  General animal: susceptible-infected-Brucella | Stochastic threshold dynamics results; Stochastic perturbation is contribute to extinction of the disease to some extent by numerical simulations. |
| Nie et al., [2022](#nie2022) | ODE (SIF) | Testing and culling strategies can induce complex transmission dynamics that can help us develop appropriate prevention and control measures for animal brucellosis |
| Liang et al., [2022](#Liang2022) | ODE; Ruminant (SEIV)-Cattle (SEIV)-Human(SIR) | The prevention and control measures for Brucella should be to detect infected livestock in time, reduce contact with infected livestock and increase the cure rate of infected humans. |
| Wu et al., [2022a](#wua) | DDE stage-structure  Animal | If density-dependent growth of animals is ignored, the risk of brucellosis may be far underestimated |
| Wu et al., [2022b](#wub) | DDE multi delay | Killing sick animals may be the most effective means; Elimination of infected animals, disinfection of the environment, vaccination and education of people are the effective prevention and control strategies |
| Zeng et al., [2022](#zeng2022) | SDE  Susceptible-aborting infected-infectious carrier | Environmental noise can be helpful to the extinction of the disease in the flock and decaying of the Brucella in the environment |
| Liu et al., [2023](#Liu) | A reaction-diffusion with seasonality, spatial heterogeneity and nonlocal delay  SLICB (susceptible-latent-acute infected-chronic infected-brucella) | Extending the latent period and increasing the random walk rate of infected sheep can effectively prevent brucellosis from developing into an endemic disease; The greater the density of acute infections, the higher the risk of brucellosis transmission, and the density of infected sheep and the time to reach the stable state will have a large deviation if only consider acute or chronic sheep; Reaching the peak time will be delayed if the peak time of sheep birth is delayed. |
| Wang et al., [2023](#wang2023b)b | SDE  Susceptible young sheep-susceptible adult or sexually mature sheep-vaccinated-infectious  brucella in the environment | Threshold Dynamics; Environmental white noise contributes to the extinction of brucellosis to a certain extent |

**Table A2: Overview of the articles on applied researches included in this review.**

| Literature | Data | Data source | Model structure |
| --- | --- | --- | --- |
| Hou et al., [2013](#Hou2013) | 2005-2010 human cases | Bulletin of China’s major zoonotic epidemics | Cont. ODE; Constant input rate; Mass action incidence rate  Sheep(SEIV)-Human(SAC)-Brucella |
| Zhang et al., [2014a](#zhang2014a) | 2001 to 2010 infectious dairy cows | Zhejiang Province | Cont. SE; Dairy Cattle(SEI)-Brucella |
| Li et al., [2014b](#Li2014b) | human brucellosis data from 2001 to 2011 | Epidemiological bulletins published by Hinggan League centers for disease control and prevention. | Cont. ODE; Other sheep(SRiQSiV)-Basic ewes(SRiQSiV)-Human(SAC)-Brucella |
| Nie et al., [2014](#nie2014) | Dairy Cattle in Jilin province 1987- 2005 | Animal epidemic prevention and control center of Jilin province | Cont. ODE; Dairy cows(SEI)-Bruce |
| Hou et al., [2015](#Hou2015) | Positive rate of adult sheep from 2008-2012 | China Animal Health and Epidemiology Center | Cont. ODE; Adult(young)Sheep(SVEI) |
| Zhang et al., [2015](#zhang2015) | Monthly human cases from 2004 to 2011 in Hinggan League | - | Non-autonomous Cont. ODE; Sheep(SEI)-Human(SEI)-Brucella |
| Lou et al., [2016](#Lou) | Newly acute human brucellosis cases from 2010 to 2014 | Center for Disease Control and Prevention of Bayingolin Mongol Autonomous Prefecture in Xinjiang | Cont. ODE;Sheep/cattle(SEIV)-Human(SAC) |
| Li et al., [2017a](#Li2017a) | 2004-2014 human cases in China | National Notifiable Disease Surveillance System | Cont. ODE; Sheep(SI)-Human: (SEAC)-Brucella |
| Zhang et al., [2017](#zhang2017) | 2000-2013 human cases in Jilin Province | Public health science data center | Cont. ODE; Sheep(SI)-Environment brucellosis |
| Wang et al., [2018b](#wang2018b) | 2010-2014 human cases by seasons | Public health science data center | Non-autonomous SDE; Sheep/cattle(SEIV)- Human(SAC) |
| Zhou et al., [2018](#zhou2018) | 2001-2010 reported sheep brucellosis and human cases in Inner Mongolia | Regions of Inner Mongolia | Cont. ODE and optimal control model; Sheep(SIV)-High-risk human(SAC)-low-risk human(SAC) |
| Peng et al., [2020](#peng2022b)b | Human brucellosis incidence between 2015 and 2018  Epidemic data of cattle and sheep in China between 2005 and 2016 | Data Center of the China Public Health website&  A research study published in Chinese journal | ODE; Constant input rate; Mass action  Young Sheep(SE)-adult Sheep(SE)-vaccinated Sheep-Young Cattle(SE)-adult Cattle(SE)-vaccinated Cattle-Human(SAC) |
| Guo et al., [2022](#Guo) | Cumulative new human cases from 2010 to 2020 in Jalaid banner &Ulanhot | Public health science data center | Cont. ODE considered transportation; Sheep(SEIV)-Human(SEI)-Brucella |
| Ma et al., [2022a](#ma2022a) | 2016 to 2020 human cases in Inner Mongolia | National Notifiable Disease Report System | Non-autonomous cont. ODE; Sheep(SAiQV)-Human(SAC)-Brucella |
| Ma et al., [2022b](#ma2022b) | 2005-2020 human cases in Jilin | Public health science data center | Discrete ODE ODE; Sheep(SAI)-Human(SAC)-Brucella |
| Ma et al., [2024](#ma2022b) | 2005-2020 human cases in Xinjiang | Xinjiang Uygar Autonomous Region by China CDC | DDE; Sheep(SEIQV)-Human(SAC)-Brucella |
| Qin et al., [2022](#Qin) | Human brucellosis in Shanxi Province and Hebei Province from 2010 to 2020 | _ | ODE; Sheep(SI)-Human(SAC)-Brucella |
| Gong et al., [2023](#gong) | Number of reported brucellosis cases in Ningxia from 2005 to 2021 | Infectious Disease Reporting Information Management System of Ningxia Center for Disease Control and Prevention | Sheep: susceptible lambs-susceptible adult-latent-infected- immunodetection  Human: susceptible-acute infectious-chronic infectious -environment |
| Wang et al., [2023a](#wang2023) | Price of sheep’s live weight and the cumulative cases of human brucellosis from 2015 to 2020 in Ningxia | National Notifiable Diseases Surveillance System  Compilation of Cost-benefit Data of National Agricultural Products | Susceptible-infected- immune-environment-price-human |
| Wang et al., [2023c](#wang2023) | Sheep price data and infections for Henan 2016~2021 | - | Susceptible-infected-environment-price-inventory-human |
| Yue et al., [2023](#yue2023) | Sheep data from Yulin 2005-2014 | Statistical bulletin of national economic and social development of Yulin City | Susceptible young sheep-susceptible adult sheep-immune sheep-infected sheep-Brucella |
| Zhang et al., [2023](#zhang2023) | Human brucellosis in Inner Mongolia from 2016 to 2020; Meteorological factors | China Center for Disease Control and Prevention | Non-autonomous cont. ODE  Sheep: susceptible sheep (S), exposed sheep (E) and infected sheep  Human: susceptible-infected-chronic infections  Brucella |
|  |  |  |  |

**Reference**

1. L. Dang, X. Abdurahman, and Z. Teng, “The Threshold Dynamics of a Stochastic Two-Patch Brucellosis,” Stochastic Models, vol. 38, no. 3, pp. 331–364, 2022.
2. Q. Hou, X. Sun, Y. Wang, B. Huang, and Z. Jin, “Global Properties of a General Dynamic Model for Animal Diseases: A Case Study of Brucellosis and Tuberculosis Transmission,” Journal of Mathematical Analysis and Applications, vol. 414, no. 1, pp. 424–433, 2014.
3. Q. Hou, “Global Analysis of a Multi-Group Animal Epidemic Model With Indirect Infection and Time Delay,” Journal of Applied Analysis & Computation, vol. 6, no. 4, pp. 1023–1040, 2016.
4. Q. Hou and F. Zhang, “Global Dynamics of a General Brucellosis Model With Discrete Delay,” Journal of Applied Analysis & Computation, vol. 6, no. 1, pp. 227–241, 2016.
5. Q. Hou and H. Qin, “Global Dynamics of a Multi-Stage Brucellosis Model With Distributed Delays and Indirect Transmission,” vol. 16, no. 4, pp. 3111–3129, 2019.
6. Q. Hou, L. Zhang, and M. Liu, “Mathematical Analysis of a Time-Delayed Model on Brucellosis Transmission With Disease Testing Information,” International Journal of Biomathematics, vol. 13, no. 5, Article ID 2050039, 2020.
7. Y. Huang and M. Li, “Optimization of Precontrol Methods and Analysis of a Dynamic Model for Brucellosis: Model Development and Validation,” JMIR Medical Informatics, vol. 8, no. 5, Article ID e18664, 2020.
8. C. Li, Z. G. Guo, and Z. Y. Zhang, “Transmission Dynamics of a Brucellosis Model: Basic Reproduction Number and Global Analysis,” Chaos, Solitons & Fractals, vol. 104, pp. 161–172, 2017.
9. M. Li, X. Pei, J. Zhang, and L. Li, “Asymptotic Analysis of Endemic Equilibrium to a Brucellosis Model,” Mathematical Biosciences and Engineering, vol. 16, no. 5, pp. 5836–5850, 2019.
10. J. Liang, Z. Zhao, and C. Li, “Rich Dynamics of a Brucellosis Model With Transport,” Complexity, vol. 2020, Article ID 5050393, 7 pages, 2020.
11. G. Liang and H. Fang, “Transmission Dynamics of a Brucellosis Model With Incubation Period,” International Journal of Biomathematics, vol. 15, no. 5, Article ID 2250024, 2022.
12. S. M. Liu, Z. Bai, and G. Q. Sun, “Global Dynamics of a Reaction-Diffusion Brucellosis Model With Spatiotemporal Heterogeneity and Nonlocal Delay,” Nonlinearity, vol. 36, no. 11, pp. 5699–5730, 2023.
13. L. F. Nie, F. Zhang, and L. Hu, “Nonlinear State-Dependent Pulse Control for an SIRS Epidemic Model with Varying Size and Its Application to the Transmission of Brucellosis.,” Mathematical Modelling of Natural Phenomena, vol. 16, p. 58, 2021.
14. Y. Nie, X. Sun, H. Hu, and Q. Hou, “Bifurcation Analysis of a Sheep Brucellosis Model With Testing and Saturated Culling Rate,” Mathematical Biosciences and Engineering, vol. 20, no. 1, pp. 1519–1537, 2022.
15. W. Wang and X. Abdurahman, “Dynamics of a Stochastic Multi-Stage Sheep Brucellosis Model With Incomplete Immunity,” International Journal of Biomathematics, vol. 16, no. 8, 2023.
16. H. Wu, W. Chen, N. Wang, L. Zhang, H.-L. Li, and Z. Teng, “A Delayed Stage-Structure Brucellosis Model With Interaction among Seasonality, Time-Varying Incubation and Density-Dependent Growth,” International Journal of Biomathematics, vol. 16, no. 6, 2022.
17. M. Wu, X. Abdurahman, and Z. Teng, “Optimal Control Strategy Analysis for an Human-Animal Brucellosis Infection Model With Multiple Delays,” Heliyon, vol. 8, no. 12, p. e12274, 2022.
18. C. Yang, P. O. Lolika, S. Mushayabasa, and J. Wang, “Modeling the Spatiotemporal Variations in Brucellosis Transmission. Nonlinear Analysis: Real World Applications,” Threshold Dynamics of an Age-Space Structured Brucellosis Disease Model With Neumann Boundary Condition. Nonlinear Analysis: Real World Applications, vol. 50, pp. 192–217, 2017.
19. J. Yang, R. Xu, and H. Sun, “Dynamics of a Seasonal Brucellosis Disease Model with Nonlocal Transmission and Spatial Diffusion,” Communications in Nonlinear Science and Numerical Simulation, vol. 94, 2020.
20. G. Zeng and X. Abdurahman, “Stationary Distribution and Extinction of a Stochastic Cattle Brucellosis Model,” Results in Applied Mathematics, vol. 15, Article ID 100320, 2022.
21. W. Zhang, J. Zhang, Y.-P. Wu, and L. Li, “Dynamical Analysis of the SEIB Model for Brucellosis Transmission to the Dairy Cows With Immunological Threshold,” Complexity, vol. 2019, no. 1, Article ID 6526589, 13 pages, 2019.
